# Chemical Heterogeneity Assessment of Authentic Edible Bird’s Nests Using Multimodal FTIR Spectroscopy: A Foundation for Future Authentication Strategies

**DOI:** 10.3390/s26051491

**Published:** 2026-02-27

**Authors:** Dung Manh Ho, Agnieszka M. Banas, Krzysztof Banas, Utkarsh Mali, Mark B. H. Breese

**Affiliations:** 1 Center for Nuclear Technologies (CNT-VINATOM), 217 Nguyen Trai Street, Cau Ong Lanh Ward, Ho Chi Minh City 71014, Vietnam; dunghm_cnt@mic.gov.vn; 2 Singapore Synchrotron Light Source, National University of Singapore, 5 Research Link, Singapore 117603, Singapore; mark.breese@nus.edu.sg; 3 Department of Physics, University of Toronto, Toronto, ON M5S 1A7, Canada; utkarsh.mali@utoronto.ca

**Keywords:** edible bird’s nest authentication, FTIR spectroscopy, ATR-FTIR, micro-ATR imaging, chemical heterogeneity, Principal Component Analysis (PCA), chemometric analysis

## Abstract

**Highlights:**

**What are the main findings?**

- Micro-ATR imaging provides unique insights into spatial compositional variations that are masked in bulk FTIR measurements.
- Mean or single-point spectra do not adequately capture the chemical complexity of genuine EBN.

**What are the implications of the main findings?**

- Authentication strategies should move beyond single-spectrum matching toward library-based and statistically informed FTIR models.
- Multi-point and imaging-enabled measurements are essential for distinguishing intrinsic variability from compositional anomalies in EBN.

**Abstract:**

Edible Bird’s Nest (EBN) is a highly prized food product, making it a frequent target for economic adulteration. Consequently, robust quality assurance is paramount to protect consumers and ensure market integrity. A significant barrier to effective quality control, however, is an incomplete understanding of the natural chemical variability within authentic EBN. This variability, influenced by factors such as geographical origin, bird species, and post-harvest processing, can confound analytical measurements and complicate the definition of a standard reference. This study provides an existence proof in a defined cohort, characterizing microscale chemical heterogeneity in authentic *A. fuciphagus* EBN. We employed a multi-modal Fourier Transform Infrared (FTIR) spectroscopy approach, integrating transmission, macro-attenuated total reflectance (ATR), and high-resolution micro-ATR chemical imaging. A diverse set of validated, authentic EBN samples was analyzed using unsupervised Principal Component Analysis (PCA) to explore the data structure. Our results reveal significant and previously unquantified spectral heterogeneity, particularly in protein and glycoprotein-related regions. In our cohort, the chemical signatures of authentic EBN do not collapse to a single, uniform profile but span a broad, multi-dimensional continuum. This inherent variability presents a critical challenge for conventional quality control methods that rely on simplistic, single-spectrum standards, which may lead to the misclassification of genuine products. By establishing a robust chemical baseline for the authentic class, this work provides the foundational data essential for developing next-generation authentication models capable of reliably distinguishing this natural variance from deliberate adulteration.

## 1. Introduction

Edible Bird’s Nest (EBN) is a high-value natural bioproduct formed from the salivary secretions of swiftlets distributed around the Indian Ocean and the South China Sea. It has been consumed for over five centuries in traditional Chinese cuisine and medicine, where it is regarded as a luxury food comparable to caviar or foie gras [1,2,3]. Historical records trace its use to the Ming Dynasty, while in the modern market EBN may reach prices exceeding USD 4000 per kilogram, driven by cultural prestige and purported health benefits such as enhanced bone strength, cell regeneration, anti-aging effects, and antiviral activity [1,2,4,5,6,7,8,9,10].

To meet increasing demand, EBN production has shifted from traditional cave harvesting to large-scale artificial swiftlet farming [1,11,12]. Despite improved supply stability, the exceptionally high economic value of EBN has made it particularly susceptible to food fraud. Common adulteration practices include the addition of lower-cost materials such as tremella fungus, gelatin, karaya gum, or seaweed, often combined with staining or bleaching to conceal foreign substances [2,4,13,14]. Consequently, reliable authentication and quality control of EBN have become critical concerns for consumers, regulators, and the food industry [4,13,15].

A wide range of analytical approaches has been proposed for EBN characterization and authentication, including protein profiling, chromatography, elemental analysis, electron microscopy, and spectroscopic techniques [2,4,13,15]. Among these, Fourier Transform Infrared (FTIR) spectroscopy has attracted significant attention owing to its non-destructive nature, low operational cost, and ability to generate molecular fingerprint information [16,17,18,19,20]. FTIR-based methods are therefore frequently promoted as rapid tools for distinguishing authentic EBN from adulterated products.

However, a closer examination of the literature reveals notable methodological limitations. Many studies rely on visual inspection of a limited number of spectra, often acquired from few sampling points and with insufficient spectral quality or statistical power [16,17,19,20]. Transmission-mode FTIR measurements using KBr pellets inherently average chemical composition and eliminate spatial information, while ATR-based studies frequently draw qualitative conclusions without rigorous statistical validation [16,19,20]. Even when chemometric tools such as Principal Component Analysis (PCA) are employed, they are often applied to datasets that are too small to capture the intrinsic variability of a complex biological material such as EBN [17,19,20].

More critically, much of the existing work implicitly assumes that authentic EBN exhibits a chemically uniform composition. In reality, EBN is a natural glycoprotein-rich biopolymer whose chemical characteristics are influenced by multiple factors, including swiftlet species, geographic origin, diet, environmental conditions, and post-harvest processing [4]. While general spectral similarities among EBN samples are widely reported, systematic characterization of intra-sample chemical heterogeneity, particularly using spatially resolved spectroscopic techniques, remains largely unexplored. This gap raises a fundamental question regarding the validity of representing authentic EBN with a single or narrowly defined spectral reference.

Current spectroscopic sensors for EBN often operate under the flawed assumption of chemical uniformity, which leads to narrow reference libraries and high false-negative rates [21]. By transitioning from a ’point source’ sensing paradigm to a spatially resolved micro-ATR imaging approach, this work deconstructs the stochastic distribution of biochemical components, providing the necessary high-resolution data to refine sensing algorithms for complex natural biopolymers. Without a rigorous definition of the chemical space occupied by authentic EBN, authentication strategies based on limited spectral benchmarks are inherently vulnerable to misclassification of genuine samples, leading to false positives and reduced analytical reliability. Therefore, a prerequisite for robust authentication is a comprehensive understanding of the intrinsic chemical variability of authentic EBN.

In this study, we address this foundational issue by systematically characterizing the chemical heterogeneity of validated authentic EBN samples using a multimodal FTIR framework. Transmission FTIR spectroscopy, macro-ATR measurements, and, for the first time in this context, high-resolution micro-ATR chemical imaging are combined to investigate both bulk and spatially resolved molecular features. By quantifying intra- and inter-sample variability, this work establishes a robust chemical baseline for authentic EBN, providing a critical foundation for the development of more reliable FTIR-based authentication strategies. Importantly, while we demonstrate that authentic EBN is intrinsically heterogeneous, we do not treat the presence of heterogeneity as an authenticity criterion. Rather, we establish the authentic class’s spectral baseline and spatial variance to enable future discriminative models that must separate intrinsic variability from adulteration. We emphasize that the present work is hypothesis-generating and bounded to the sampled cohort; establishing population-level prevalence or stability of any spectral attribute requires substantially larger, multi-species and multi-site studies.

## 2. Materials and Methods

### 2.1. Sample Collection and Preparation

**Figure 1:**
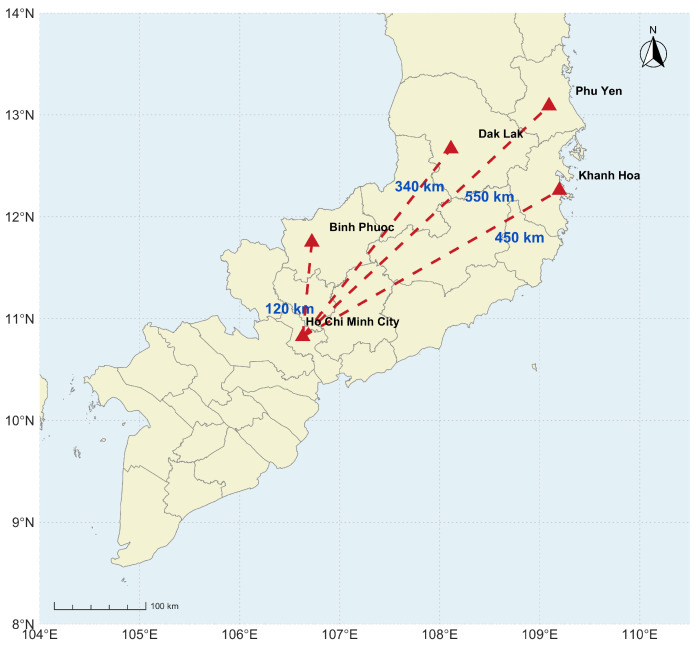
Spatial distribution and distances from Ho Chi Minh City to provinces in Southern and Central Vietnam. Dashed lines indicate the directions to Binh Phuoc (120 km), Dak Lak (340 km), Phu Yen (550 km), and Khanh Hoa (450 km).

To interrogate the intrinsic biological and chemical variability of edible bird’s nest, six raw EBN samples were selected from caves across four geographically distinct provinces in Vietnam: Dak Lak (DL), Phu Yen (PY), Khanh Hoa (SA, MN, NH), and Binh Phuoc (BP) what is presented in Figure 1. These sites span diverse ecological zones, from southern highlands to central coasts. These provinces are Vietnam’s leading EBN production hubs, with over 29,000 swiftlet houses nationwide yielding >150 tons annually, valued at >USD 600 million. Exports exceeded USD 8 million in 2024, up from USD 6 million in 2023, with key markets like China and the US. Khanh Hoa is the “capital” of the industry, while Dak Lak, Phu Yen, and Binh Phuoc are major contributors. Sourcing from these four distinct regions separated by hundreds of km ensures our six samples capture regional biological and environmental variability within Vietnam’s EBN population. This aligns with our study’s focus on intrinsic heterogeneity as a baseline for future authentication.

The samples were received in their native fragmented form (flakes), packaged in zip-lock bags, and stored at ambient temperature (25 °C) prior to analysis. Before spectroscopic characterization, each specimen was systematically examined under optical magnification, and residual feathers or extraneous particulate matter were carefully removed using fine tweezers. This minimal yet rigorous cleaning protocol was designed to ensure compositional purity while preserving the fragile, native microstructure of the EBN material. The samples were collected from nests produced by the White-nest swiftlet (*Aerodramus fuciphagus*), the primary species utilized for commercial EBN production in Southeast Asia. This specificity ensures that the observed heterogeneity is an intrinsic characteristic of this species’ secretions rather than an artifact of inter-species variation. We emphasize that the six nests analyzed constitute six biological replicates. All repeated measurements per nest are technical replicates used to resolve intra-sample chemical variability. Pixel spectra are not considered biological replicates.

For analysis, EBN samples were prepared as small flakes, typically a few millimeters in size, with a maximum lateral dimension of approximately 5 mm. Although the samples exhibited comparable macroscopic morphology, subtle yet consistent visual differences were observed among geographic origins, particularly in coloration ranging from off-white to pale yellow. Such variations are consistent with natural variability reported in previous studies and are commonly attributed to differences in swiftlet diet, nesting environment, and secretion chemistry. Importantly, the sampling strategy was deliberately conceived as a proof-of-concept study to test a central hypothesis: that authentic EBN is inherently chemically heterogeneous at multiple length scales. Rather than aiming for population-wide statistical representativeness, which would require a substantially larger and more geographically extensive cohort, the study was designed to capture a representative spectrum of intrinsic compositional variability within a limited number of well-defined case studies. Accordingly, all conclusions are restricted to authentic *A. fuciphagus* EBN within this cohort and are not asserted as population-level generalizations.

### 2.2. FTIR Instrumentation

All spectroscopic analyses were performed at the ISMI (Infrared Spectro-Microscopy) beamline at the Singapore Synchrotron Light Source (SSLS). The instrumental setup consisted of a Bruker Optics GmbH & Co. KG Ettlingen, Germany Vertex 80v spectrometer coupled to a Bruker Hyperion 3000 IR microscope equipped with a 128 by 128 pixels Focal Plane Array (FPA) detector. The system was configured for analysis in three distinct modes:

- Transmission Mode: for bulk analysis using standard KBr pellets.
- Macro-ATR Mode: utilizing a PIKE MIRacle™ single-reflection ATR accessory with a ZnSe/diamond crystal (1.6 mm diameter) for rapid surface analysis.
- Micro-ATR Imaging Mode: employing a dedicated 20× magnification ATR objective with an internal Germanium (Ge) crystal coupled with FPA detector for high-resolution chemical mapping.

### 2.3. FTIR Analytical Procedures

A multi-modal analytical strategy was designed to characterize the EBN samples across different scales, directly comparing conventional bulk analysis with high-resolution imaging to address the central research question of spectral heterogeneity.

#### 2.3.1. Transmission Spectroscopy (Bulk Analysis)

To replicate conventional, spatially averaged analysis, approximately 1 g of each EBN sample was pulverized using a pestle and mortar. The resulting powder was mixed with potassium bromide (KBr) and pressed into a thin pellet (13 mm diameter). To ensure repeatability, pellets were prepared in triplicate for each sample. Spectra were collected in transmission mode over the 4000–450 cm^−1^ range. For data reliability, three different spots on each pellet were analysed, with each final spectrum being an average of 65 co-added scans. This method provides a single, bulk chemical profile representing the average composition of the sample.

#### 2.3.2. Macro-ATR Spectroscopy (Surface Analysis)

For rapid, non-destructive chemical fingerprinting of intact nest fragments, spectra were acquired in macro-ATR mode. Flatter EBN fragments were selected to ensure optimal contact with the ATR crystal. For each sample, multiple spectra were collected from different areas over the 4000–525 cm^−1^ range, with each spectrum representing an average of 65 co-added scans. This approach provides a surface-sensitive analysis of the material in its native state.

#### 2.3.3. Micro-ATR Chemical Imaging (Heterogeneity Analysis)

To directly visualize the spatial distribution of chemical components and assess sample homogeneity, high-resolution imaging was performed using the 20× Ge-ATR objective coupled with the FPA detector (128 × 128). To enhance the Signal-to-Noise Ratio (SNR), 2 by 2 pixel binning was employed. This setup allowed for the simultaneous collection of 4096 spectra across a defined area of an intact EBN fragment. Each chemical map was generated from 256 co-added scans over a 15-min period of experiment, covering the spectral range of 3600–900 cm^−1^. This technique provides a detailed chemical image revealing spatial variations in protein, carbohydrate, and lipid content. Micro-ATR pixel spectra are analyzed to delineate spatially localized chemical domains within a single biological unit; they are not used to estimate between nest biological variation.

### 2.4. Spectral Acquisition and Processing

For all analytical modes, background spectra were collected prior to sample analysis (a clean ATR crystal for ATR modes; an empty sample chamber for the transmission mode). Data recording and initial processing were conducted using OPUS software (Version 7.8, Bruker, 2016). The conversion from interferogram to spectrum was performed using a Blackman-Harris 3-term apodization function, a phase resolution of 32, power spectrum phase correction mode, and a zero filling factor of 2. Following export from OPUS, further spectral processing and data organization were performed in R environment for statistical analysis and RStudio version 2025.05.1 as Graphic User Interface (GUI) [22]. In evaluating spectroscopic data, hyperSpec package [23] served as the foundation for ingesting and structuring spectra into a consistent object model, enabling wavenumber-aware operations and metadata management. It was used for essential pre-processing steps—such as baseline correction, smoothing, normalization, and interpolation—so that downstream chemometric analyses were comparable and robust across instruments and samples. ChemoSpec [24] then provided a streamlined workflow for exploratory chemometrics: generating quality control diagnostics and performing hierarchical clustering to identify sample groupings. The dendextend package [25] was applied to refine and interpret hierarchical clustering outputs, allowing us to adjust linkage methods, prune branches, color-coded clusters, and compare dendrograms across preprocessing regimes or distance metrics, thereby improving the reliability and readability of cluster assignments derived from spectral similarity. For multivariate modelling beyond exploratory steps, FactoMineR [26] was used to conduct rigorous dimension reduction (PCA) for the outlier removal procedure. To support large-scale data sets, bigstatsr [27] enabled memory-efficient handling of high-dimensional spectral matrices via file-backed matrices and scalable decompositions, ensuring that PCA could be performed without exhausting RAM. Together, this toolchain delivered a reproducible pipeline: hyperSpec for data handling and preprocessing; ChemoSpec for chemometric exploration; dendextend for robust clustering interpretation; FactoMineR for comprehensive multivariate analysis; and bigstatsr for computational scalability on large spectroscopic datasets obtained for micro-ATR mode.

## 3. Results and Discussions

### 3.1. Effect of FTIR Measurement Mode on Edible Bird’s Nest Spectra

Before a comparative analysis of the different EBN samples could be undertaken, it was essential to first establish how the choice of analytical mode: transmission, macro-ATR, or micro-ATR, fundamentally influences the resulting spectrum. The physical principles underlying these modes dictate that they probe the sample in different ways. Transmission mode measures the absorbance of infrared light as it passes through the entire volume of the sample. In this study, this required pulverizing the EBN, thereby destroying its native structure to create a homogenized powder. The resulting spectrum, therefore, represents the average bulk chemical composition of the sample, providing a comprehensive chemical profile but losing all information related to spatial heterogeneity or surface-specific chemistry. These observations establish existence within our cohort; they are not intended to estimate prevalence or stability across populations.

In contrast, both macro- and micro-ATR are surface-sensitive techniques where an evanescent wave penetrates only a few micrometres into the sample. Macro-ATR provides a chemical fingerprint of the bulk surface, while micro-ATR imaging reveals the chemical composition of micro-domains, assessing heterogeneity.

To enable a meaningful comparison between spectra obtained from these physically distinct modes, a standardized data processing workflow was imperative. All collected spectra are presented in absorbance units. A critical step for a valid comparison is the application of an ATR correction to the spectra obtained in both macro- and micro-ATR modes. This mathematical correction is necessary because the penetration depth of the evanescent wave is wavelength-dependent, causing a distortion where absorbance bands at lower wavenumbers appear artificially intense relative to those at higher wavenumbers. The correction algorithm compensates for this effect, transforming the ATR spectrum to be more directly comparable to a true transmission spectrum. By applying this correction and presenting all data in standardized absorbance units, we created a unified data set where spectral differences can be confidently attributed to genuine chemical variations rather than instrumental artifacts. This harmonization is a crucial prerequisite for any robust comparative analysis. Following this pre-processing, which also included baseline correction and area normalization, the spectra were ready for direct comparison.

**Figure 2:**
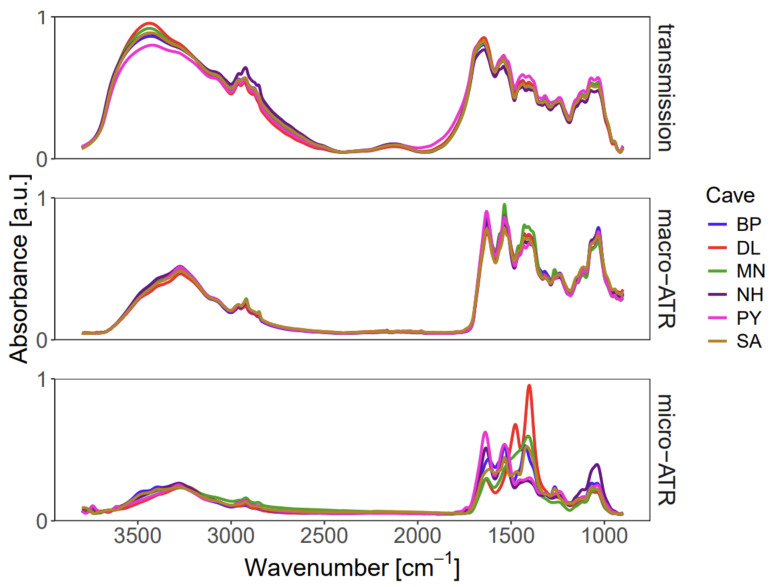
Comparison of mean FTIR spectra from six distinct edible bird’s nest (EBN) samples (each collected from a different cave) acquired using three different analytical modes: transmission (bulk analysis), macro-ATR (surface analysis), and micro-ATR (micro-domain analysis).

Figure 2 presents pre-processed FTIR spectra enabling direct comparison of the mean profiles from six EBN samples, each collected from a different cave, measured using three complementary analytical modes: transmission (bulk), macro-ATR (surface), and micro-ATR (micro-domain). To harmonize differences across instruments and sampling geometries, all spectra were consistently truncated to the 3800–900 cm^−1^ range. The subsequent analysis examines key similarities and divergences among the bulk, surface, and micro-domain chemical signatures to evaluate the extent to which any single mode can capture the overall spectral characteristics of EBN. In particular, we assess whether one analytical method can produce a spectrum that is representative of the material as a whole, or whether multi-modal measurements are required to account for intrinsic chemical heterogeneity at different length scales.

All collected spectra, regardless of the analytical mode, exhibit the prominent absorption features characteristic of EBN’s biochemical composition, as previously reported [16,17,19,20]. The dominant proteinaceous nature is confirmed by the presence of the Amide I band (C=O stretching, 1695–1630 cm^−1^) and the Amide II band (N–H bending, 1560–1500 cm^−1^). Further contributions from amino acids are indicated by absorption bands in the 1250–1020 cm^−1^ range (C–N stretching) and 1440–1395 cm^−1^ range (O–H bending of carboxylic acids). Carbohydrate moieties are observable through C–H bending at 1390–1380 cm^−1^ and C–O stretching at 1085–1050 cm^−1^. Finally, lipid content is represented by the asymmetric and symmetric stretching vibrations of CH2 groups in the 3000–2800 cm^−1^ region.

A central premise of this study is to test the hypothesis of EBN homogeneity. If EBN were a chemically homogeneous material, as has been implicitly assumed in previous studies [16,17,19,20], the FTIR spectra should exhibit a consistent pattern regardless of the analytical mode or sampling area. As depicted in Figure 2, a high degree of similarity is indeed observed between the mean spectra acquired in transmission and macro-ATR modes. This is expected, as both methods analyse a relatively large area, providing a chemical signature that represents the average bulk or surface composition.

However, significantly higher variance is observed in the spectra obtained via micro-ATR, with the standard deviation approximately doubling compared to transmission mode results, directly challenging the assumption of homogeneity. This is most strikingly illustrated by the appearance of a distinct C=O stretching vibration of triglycerides at 1745 cm^−1^, a band that is solely observed in certain micro-ATR spectra and is completely absent in the averaged bulk spectra. This finding indicates the presence of localized, lipid-rich micro-domains within the EBN structure. This specific lipid domain, characterized by the 1745 cm^−1^ peak, may reflect residual components from the swiftlet’s diet or specific secretory processes, and its isolation by micro-ATR emphasizes how natural, non-proteinaceous materials are heterogeneously incorporated into the nest matrix. The discrepancy between the modes is therefore attributed to the scale of the analysis.

**Figure 3:**
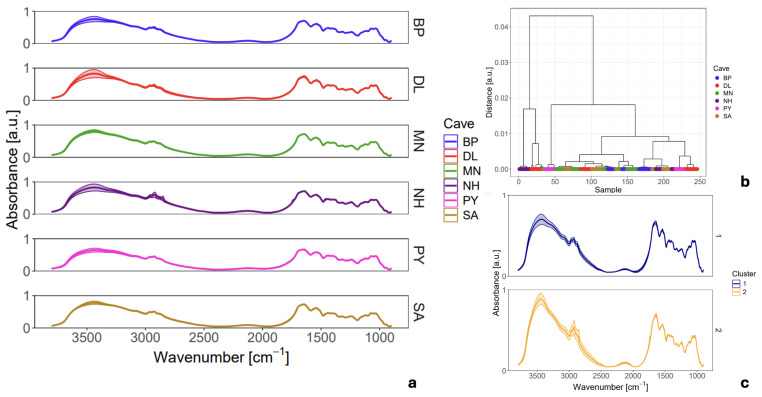
Mean FTIR spectra of raw EBN samples from six caves in four Vietnamese provinces collected in transmission mode. Each panel shows the cave-specific mean spectrum with its corresponding standard deviation (SD). The SD closely overlaps with the mean due to low spectral variability within each dataset (**a**). Dendrogram clustering of different groups of EBNs using complete linkage and Person distance (**b**). Comparison of two clusters representative spectra found at distance level 0.02 in the dendrogram (**c**).

**Figure 4:**
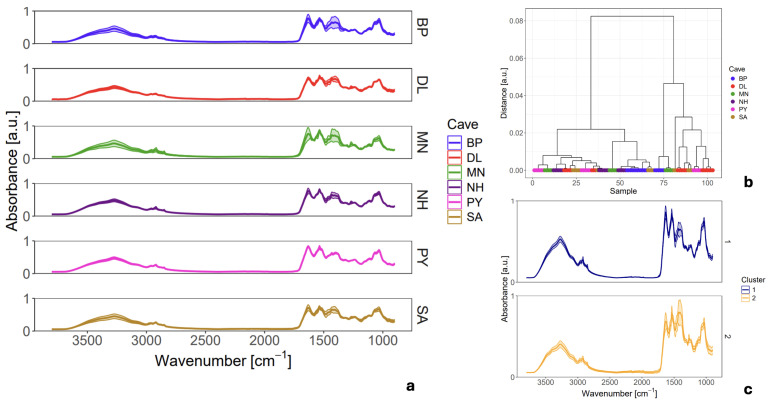
Mean macro-ATR FTIR spectra of raw EBN samples from six caves in four Vietnamese provinces. Each panel shows the cave-specific mean spectrum with overlaid standard deviation (SD), illustrating intra-sample chemical heterogeneity inherent to macro-ATR measurements (**a**). Dendrogram clustering of different groups of EBNs using complete linkage and Pearson distance (**b**). Comparison of two clusters representative spectra found at distance level 0.05 in the dendrogram (**c**).

To quantitatively assess the observed variability, for each analytical mode we plotted the mean FTIR spectrum of six distinct EBN samples, each collected from a different cave, together with the corresponding ± standard deviation (SD), as shown in Figure 3a and Figure 4a. While individual wavenumbers in a spectrum do not follow a normal distribution, the SD serves as a powerful tool to visually identify spectral regions with high variance among the collected spectra. It is important to note that this representation is used to highlight variability rather than for strict statistical inference, and it cannot identify specific outliers. The subsequent analysis will utilize these plots to pinpoint the exact biochemical components contributing to the heterogeneity of EBN.

### 3.2. Bulk Chemical Analysis via Transmission Spectroscopy: An Assessment of Homogeneity

Analysis of the homogenized EBN samples via transmission spectroscopy was performed on a total of 246 spectra acquired from the six EBN variants prepared as KBr pellets. This method, which involves pulverizing the material, inherently provides a spatially averaged, bulk chemical profile.

The mean pre-processed FTIR spectra, plotted with its corresponding standard deviations (SD) in Figure 3a, reveals a high degree of spectral homogeneity across all samples. Remarkable consistency is observed in both relative peak intensities and band positions, irrespective of the geographical origin of the EBN. To objectively probe this apparent homogeneity, Hierarchical Cluster Analysis (HCA) was employed as an unsupervised classification method. The analysis was conducted on the 3800–900 cm^−1^ spectral range using a complete linkage algorithm and the Pearson distance as the similarity measure. The Pearson distance metric was selected for its invariance to scaling and baseline offsets, providing a robust similarity measure that focuses on spectral shape rather than magnitude. Complete linkage was utilized as the aggregation method to ensure the formation of tight, homogeneous clusters.

The resulting dendrogram (Figure 3b), with leaves coloured according to sample origin, shows that all spectra are interconnected at a very low height (<0.04), confirming a high degree of overall similarity. However, a closer inspection reveals the formation of two distinct sub-clusters at a distance threshold of 0.02. A comparison of the mean spectra from these two sub-clusters (Figure 3c) indicates that the spectral differences are minimal. While minor variations in the relative intensities of some absorption bands are present, the peak positions within the critical fingerprint region show excellent correspondence. This suggests that the fundamental chemical composition across all samples is nearly identical when homogenized.

Crucially, the HCA dendrogram shows that spectra from five of the six geographical origins contribute to both clusters. This demonstrates that these subtle spectral variations are not sufficient to differentiate the EBN samples based on their provenance. Therefore, the results from the transmission mode analysis support the conclusion that when EBN is homogenized, its bulk chemical profile is highly consistent, and a single, averaged spectrum can indeed be considered representative of the raw material. This finding highlights how conventional bulk analysis methods can mask the underlying heterogeneity of the material, a key point that will be contrasted with surface-sensitive techniques in the subsequent sections.

### 3.3. Surface Chemical Analysis via Macro-ATR Spectroscopy: Unveiling Heterogeneity

To investigate the chemical composition of the EBN in its native, intact state, a total of 103 spectra were acquired from the six EBN samples using macro-ATR spectroscopy. This surface-sensitive technique studies the material without the homogenization required for transmission mode, thus preserving the original surface chemistry.

In stark contrast to the transmission mode data, the mean spectra obtained by macro-ATR is accompanied by significantly larger standard deviations (SDs), as shown in Figure 4a. This high variance indicates substantial spectral differences between the individual measurement points, directly reflecting a non-uniform distribution of key biochemical components, such as glycoproteins, amino acids, and carbohydrates, across the surface of the intact EBN fragments. Although minor scan-to-scan variability is expected due to random noise, the significant differences in band intensities observed here, coupled with the high signal-to-noise ratio (SNR) of the spectra, strongly suggest genuine chemical heterogeneity rather than instrumental artifact [28,29,30,31].

This observation is quantitatively substantiated by HCA. The resulting dendrogram (Figure 4b) reveals that a linkage distance of 0.08 is required to merge all spectra into a single cluster, a value double that observed for the homogenized transmission data (0.04). This greater distance objectively confirms that the spectra collected from the surface are significantly more dissimilar to one another than those from the bulk material. Furthermore, a comparison of the mean spectra from two representative clusters (Figure 4c) shows clear differences in relative band intensities, unlike the near-identical clusters found in the transmission analysis.

Collectively, these findings demonstrate that when examined in its native state with a surface-sensitive technique, EBN presents as a chemically heterogeneous matrix. This directly refutes the notion that a single spectrum can be considered representative of the material and underscores the limitations of relying on such an approach for authentication. The apparent homogeneity observed in the transmission mode is thus revealed to be an artifact of the sample homogenization process, which averages out these critical surface variations.

### 3.4. Micro-Scale Chemical Imaging: Definitive Evidence of EBN Heterogeneity

To resolve the heterogeneity suggested by the macro-ATR analysis, we employed high-resolution micro-ATR chemical imaging. This technique, which acquires 4096 spectra per measurement area, provides the spatial and chemical resolution necessary to probe the material’s composition at the micro-scale. A total of 15 distinct areas were mapped across the six EBN samples. These hyperspectral maps quantify intra-sample heterogeneity; aggregation or clustering of pixel spectra is used to characterize micro-domains within a nest, not to inflate biological replicate count. These micro-ATR observations are used to define authentic intra-sample variance and domain structure; they are not proposed as stand-alone authenticity markers. These observations establish existence within our cohort; they are not intended to estimate prevalence or stability across populations.

**Figure 5:**
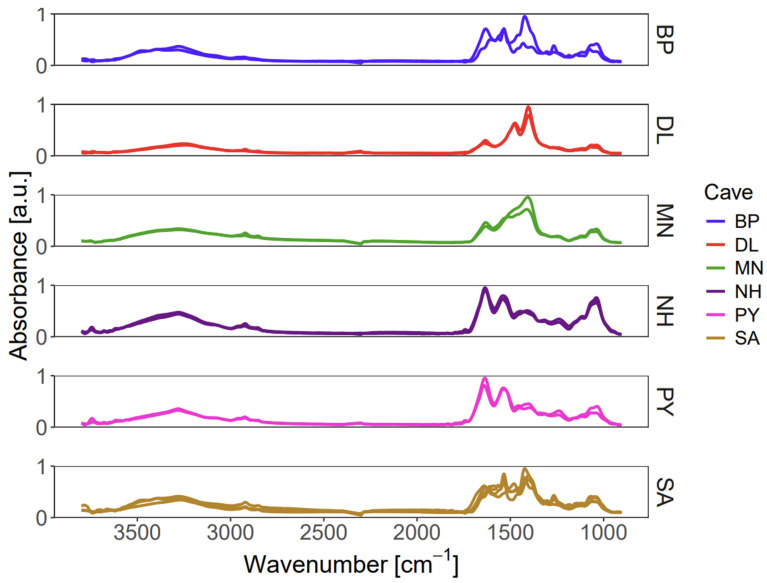
Intra-sample chemical heterogeneity of authentic EBN revealed by micro-ATR chemical imaging. Each spectrum shown is the spatially averaged mean of 4096 pixel spectra (excluding outliers) from a single mapped area. Spectra are grouped by their parent EBN sample (e.g., Cave BP). The presence of multiple, distinct spectral profiles within a single sample group demonstrates significant chemical variability across different regions of the same nest. Standard deviation envelopes are omitted for visual clarity, and intra-map variance was addressed quantitatively via MAD-based outlier removal during preprocessing.

Given the large volume of data, a multivariate statistical approach was essential. Prior to analysis, spectral outliers within each hyperspectral data cube were identified and removed using the robust Median Absolute Deviation (MAD) method, where data points exceeding three times the MAD were excluded [29]. An initial comparison of the mean spectra from each of the 15 imaging experiments revealed significant spectral diversity (Figure 5). Notably, only a minority of these spectra (6 of 15) exhibited a profile similar to that obtained via macro-ATR, providing the first direct evidence of substantial chemical variation at the micro-scale.

**Figure 6:**
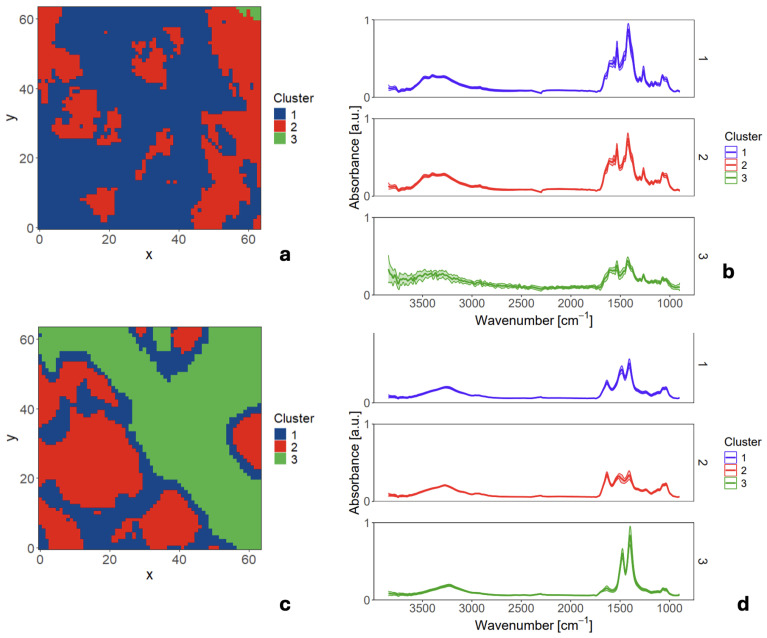
Hierarchical cluster analysis (HCA) of micro-ATR datasets: clusters are displayed as color-coded regions within the selected experimental areas, and the corresponding mean FTIR spectra for each color-coded region are shown for an EBN sample. (**a**,**b**) Example of a spectrally uniform region, where three initially selected clusters yield highly similar mean spectra. (**c**,**d**) Example of a heterogeneous region, where three clusters exhibit distinctly different mean spectra, indicating localized chemical heterogeneity across the sample surface.

To investigate the source of the observed variation, hierarchical cluster analysis (HCA) was applied to the hyperspectral micro-ATR data cube for each individual measurement area, enabling unsupervised segmentation of each mapped region into chemically distinct domains. This intra-sample analysis revealed two scenarios:

- Chemically uniform regions, in which spectra from all pixels were highly similar and collapsed into a single representative cluster (Figure 6a).
- Markedly heterogeneous regions, which segregated into two or three distinct chemical domains, each characterized by a unique mean spectrum (Figure 6b).

These clusters likely reflect an uneven surface distribution of primary biochemical components, such as localized enrichments in proteins, carbohydrates, or lipids, potentially arising from natural deposition processes and variability linked to the swiftlet’s diet.

**Figure 7:**
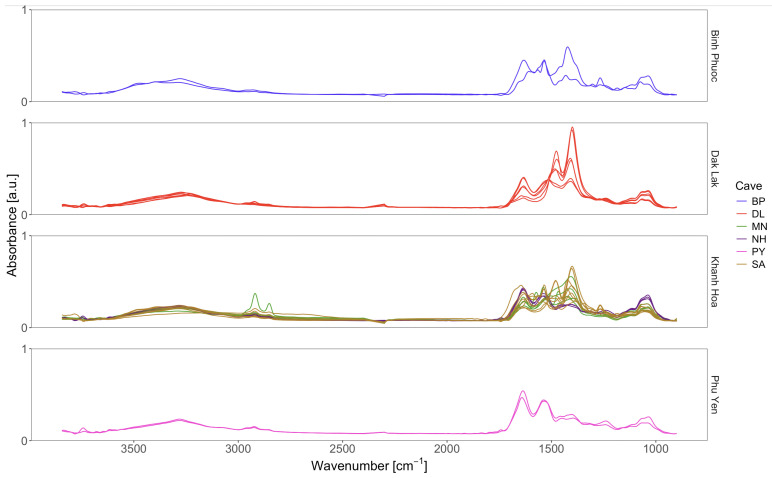
25 mini-clusters mean spectra in four panels symbolizing the four locations from which the samples were collected; colored lines were used to mark the individual sample’s membership to the particular cave.

Across all samples, the intra-sample HCA yielded 25 chemically distinct “mini-clusters.” Figure 7 presents the mean spectra of these 25 mini-clusters across four panels corresponding to the four provinces that contain the six sampled caves; colored line coding indicates each mini-cluster’s sample membership to its respective cave. To evaluate the broader micro-scale spectral landscape of EBN, these 25 mean spectra were pooled and subjected to a final, global HCA, providing an integrated view of inter-cluster relationships and quantifying the extent of chemical heterogeneity across specimens.

**Figure 8:**
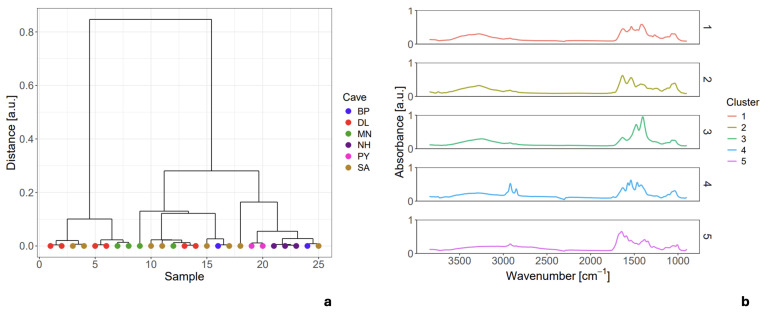
HCA of 25 mini-cluster mean spectra: dendrogram revealing three major clusters and two outliers (**a**); corresponding mean spectra for the five groups (**b**).

The resulting dendrogram (Figure 8a) definitively segregates the dataset into three major clusters and two distinct outliers. The mean spectra corresponding to these five groups are shown in Figure 8b.

This result provides conclusive evidence that EBN is not a homogeneous material and cannot be accurately characterized by a single spectrum. Crucially, none of the three primary clusters are exclusive to a single geographical origin; instead, they are composed of spectra from samples sourced across multiple caves. This demonstrates that, while significant micro-heterogeneity exists, it does not correlate with the sample’s provenance. The two outlier spectra (clusters 4 and 5) represent unique chemical compositions, possibly indicating localized contaminants or highly unusual biochemical features, further complicating the notion of a single standard spectrum. The clustering structure reported here characterizes intra-cohort diversity and should be interpreted as a baseline map for this specific sample set, pending validation in larger, more diverse collections.

Heterogeneity is expected in both authentic and adulterated multi-component materials. Discrimination will likely require ‘differential heterogeneity’ metrics such as domain size distributions, spatial co-localization among amide/carbohydrate/lipid bands, and micro-domain texture features, evaluated against a negative class. Such analyses were beyond the scope of this proof of concept and are planned as the next phase.

In summary, the micro-ATR imaging results unequivocally demonstrate that EBN is a complex, heterogeneous matrix at the micro-scale. The chemical variations observed are an intrinsic property of the material and are not linked to geographical origin, invalidating the use of a single, arbitrary spectrum for authentication purposes.

## 4. Conclusions

The primary objective of this study was to move beyond the simplistic assumptions underpinning much of the current literature and to rigorously characterize the intrinsic chemical heterogeneity of authentic Edible Bird’s Nest (EBN). The central question posed by the limitations of previous work, whether a single FTIR spectrum can meaningfully represent such a complex biological material, can now be answered. Our results, derived from a multi-modal FTIR approach, unequivocally demonstrate that authentic EBN is not chemically uniform. Instead, it exhibits significant and quantifiable heterogeneity at both the macro- and micro-scales, confirming our central hypothesis.

The Principal Component Analysis (PCA) of macro-ATR spectra from a diverse set of authentic samples revealed a wide distribution in spectral space, rather than a tight, single cluster. This finding directly challenges the validity of authentication methods that rely on comparing a sample to a single “gold standard” reference spectrum. Such an approach would inevitably fail to encompass the full scope of natural variation, leading to a high risk of false-positive classifications where genuine EBN is incorrectly flagged as non-authentic. Our data suggest that the chemical signature of “authentic EBN” is not a static point, but a dynamic, continuous space. Furthermore, our use of high-resolution micro-ATR chemical imaging, the first such application for EBN analysis, provided unprecedented insight into the spatial distribution of this heterogeneity. The chemical maps revealed distinct molecular domains within a single EBN strand, with notable variations in the relative intensities of amide I/II bands and carbohydrate-related signals. This directly refutes the implicit assumption of homogeneity made by methods like transmission FTIR with KBr pellets, which physically average out this critical spatial information. The micro-scale variability we observed proves that even a single-point ATR measurement can be misleading, as its result will depend entirely on the specific location probed on the sample surface.

The implications of these findings for the food industry and regulatory bodies are profound. Our work demonstrates that simplistic, rapid screening methods based on narrow spectral matching are fundamentally unreliable for EBN authentication. The high intra-class variance of authentic EBN means that a more sophisticated analytical strategy is required; the one that can model this variability. This study provides the essential first step in developing such a strategy by defining the boundaries of the “positive class” (i.e., authentic EBN). By establishing a robust chemical baseline that accounts for natural diversity, we provide the necessary foundation for building advanced chemometric models.

The logical and necessary next step in this line of research is to challenge this newly defined authentic baseline with a “negative class” comprising common adulterants. With a well-characterized authentic group, supervised classification models such as PLS-DA or SIMCA can be trained far more effectively. These models will be tasked not with matching a single point, but with determining whether a new sample falls within the multi-dimensional space of authenticity or outside of it, in a region occupied by adulterants. Our work, therefore, does not represent a complete authentication framework, but rather provides the indispensable data and methodological blueprint upon which truly reliable frameworks can now be built.

Our findings do not imply that heterogeneity alone authenticates EBN. Instead, they show that authentication strategies must model the authentic class’s variance and spatial organization. In the next phase, we will incorporate a negative class of common adulterants and evaluate supervised models that leverage multi-point and imaging-derived spectral features for discrimination.

In conclusion, this study has successfully provided the first comprehensive, multi-modal FTIR characterization of chemical heterogeneity in authentic Edible Bird’s Nest. We have demonstrated that genuine EBN exhibits significant intra- and inter-sample variability at both the macro- and micro-structural levels, particularly in its protein and glycoprotein composition. Within our cohort, the observed variability shows that single-spectrum comparisons are inadequate to capture authentic intra-class diversity. We therefore propose, subject to validation in expanded cohorts, that reliable authentication methods should model authentic variance and spatial organization (e.g., via multi-point/imaging and library-based approaches) rather than rely on single-reference matching. By rigorously mapping this chemical space, our study establishes the essential and previously missing foundational baseline for the “authentic” class, providing the critical data required for the future development of robust, next-generation quality control and authentication models. Our conclusions are bounded to authentic *A. fuciphagus* EBN within the sampled cohort. Population-level generalization across species, geographies, and rearing conditions will require expanded biological sampling. With the authentic chemical space defined at the micro-scale, future work will train supervised models against a negative class of common adulterants.

## Data Availability

The data presented in this study are available on request from the corresponding authors due to their size and complexity.

## Abbreviations

The following abbreviations are used in this manuscript:

EBN	Edible Bird’s Nest
FTIR	Fourier Transform Infrared
ATR	Attenuated Total Reflectance
